# Neuronal Reprogramming for Tissue Repair and Neuroregeneration

**DOI:** 10.3390/ijms21124273

**Published:** 2020-06-16

**Authors:** Roxanne Hsiang-Chi Liou, Thomas L. Edwards, Keith R. Martin, Raymond Ching-Bong Wong

**Affiliations:** 1 Centre for Eye Research Australia, Royal Victorian Eye and Ear Hospital, Melbourne, VIC 3002, Australia; hliou@student.unimelb.edu.au (R.H.-C.L.); thomas.edwards@unimelb.edu.au (T.L.E.); Keith.martin@unimelb.edu.au (K.R.M.); 2 Ophthalmology, Department of Surgery, University of Melbourne, Melbourne, VIC 3002, Australia; 3 John van Geest Centre for Brain Repair, University of Cambridge, Cambridge CB2 0PY, UK

**Keywords:** cell reprogramming, pluripotent stem cells, retina, neuroregeneration

## Abstract

Stem cell and cell reprogramming technology represent a rapidly growing field in regenerative medicine. A number of novel neural reprogramming methods have been established, using pluripotent stem cells (PSCs) or direct reprogramming, to efficiently derive specific neuronal cell types for therapeutic applications. Both in vitro and in vivo cellular reprogramming provide diverse therapeutic pathways for modeling neurological diseases and injury repair. In particular, the retina has emerged as a promising target for clinical application of regenerative medicine. Herein, we review the potential of neuronal reprogramming to develop regenerative strategy, with a particular focus on treating retinal degenerative diseases and discuss future directions and challenges in the field.

## 1. Introduction

The vertebrate retina is a multi-layer tissue at the back of the eye that is responsible for visual function. The transmission of the visual information starts with the conversion of photons energy into membrane potential changes by the photoreceptors. Within the outer nuclear layer, the rod photoreceptors are responsible for dim-light vision, while the cone photoreceptors are involved in acuity and color vision [1]. Electrical signals are transmitted from the photoreceptors to the surrounding bipolar cells which extract and convey visual signals to the retinal ganglion cells (RGCs), the output neurons of the retina. The signal transductions along this vertical pathway—from photoreceptors to bipolar cells to RGCs—are modulated by inhibitory interneurons at two different levels, horizontal cells and amacrine cells in the outer and inner plexiform layer, respectively [2,3]. Ultimately, the visual signals are transmitted by the RGCs that formed the optic nerve to the visual cortex. Given the complexity of the retina, damage to any individual neuron class within the neural retina could lead to disruption of the retinal function and vision loss. 

Pathologies of the neural retina represent some of the most common causes of vision impairment and blindness [4]. Two major retinal degenerative disorders are age-related: glaucoma, which is caused by degeneration of the optic nerve; and age-related Macular Degeneration (AMD), which is characterized by degeneration of retinal pigment epithelium (RPE) and photoreceptors. These two retinal dystrophies combine to affect 250–280 million patients worldwide [5,6]. In addition, over 200 genes have been identified to cause degeneration of different retinal cells in a number of inherited retinal disorders (IRD) [7]. For example, retinitis pigmentosa causes irreversible vision loss due to the degeneration of photoreceptors, while Leber’s hereditary optic neuropathy is an inherited mitochondrial disorder that results in degeneration of RGCs. Dominant optic atrophy is another common IRD inherited in an autosomal dominant pattern with high penetrance, which also leads to visual defects by damaging RGCs and the optic nerve. Importantly, there is no cure for these blinding diseases once the retinal cells are lost. Thus, regenerative medicine has provided an attractive approach to repair the retina and restore vision in patients. Here, we will review the current advances in using pluripotent stem cells (PSCs) and direct reprogramming approaches to generate neurons and discuss the opportunities and challenges in their application in retinal regeneration. 

## 2. Induced Pluripotent Stem Cells (iPSCs) as a Cell Source for Neuron Production

Current advances in cell reprogramming technology allow the conversion of one cell type into another, providing an attractive strategy to generate retinal neurons to repair the retina. Generally, the cell identity of a terminally differentiated cell is remarkably stable, with limited cellular potential to transform into other cell types. However, recent studies showed that forced expression of fate-determining factors can reprogram mature cells into different cell fates. In a landmark study, it was demonstrated that a single transcription factor, *MyoD*, is sufficient to convert fibroblast cells and various other cell types into skeletal muscle cells [8]. A subsequent study showed that ectopic expression of the *eyeless*, *Drosophila* homolog of *PAX6*, can induce eye development on the legs of *Drosophila* [9], demonstrating another remarkable example of cell-fate reprogramming by a single transcription factor. In addition, the seminal studies from Shinya Yamanaka’s group described a way to induce cellular pluripotency experimentally; they successfully reprogrammed adult fibroblasts to a pluripotent state with a defined cocktail of transcription factors. The resulting cells, iPSCs, are similar to embryonic stem cells (ESCs) and have the potential to differentiate into cell lineages of all three germ layers [10,11]. Importantly, iPSCs represent a non-ethically disputed and virtually infinite source of pluripotent cells [12], providing a new paradigm in regenerative medicine for a wide range of diseases. Patient-specific iPSC can be expanded and differentiated into specific neurons that are affected by the diseases, offering the unique opportunity to develop in vitro disease models for retinal diseases, including work from our group and others [13,14,15,16]. iPSC-derived neurons carrying a specific disease-related genetic background may serve as a robust platform for further investigation of pathogenetic mechanisms and reveal disease-specific cellular phenotypes. These iPSC disease models could be used for testing therapeutic interventions, such as candidate or novel drugs and neuroprotective compounds [17].

## 3. Neuronal Differentiation of iPSCs Using Small Molecules and Growth Factors

The conventional strategy of neuronal differentiation involved first directing iPSCs differentiation into neural progenitor cells (NPCs), followed by subsequent differentiation into functional neurons. Generation of NPCs and differentiated neurons from iPSCs were largely based on various animal studies of neurodevelopment, in which the major events of mammalian neural commitment are identified. During neurodevelopment, a number of signaling pathways have been implicated in the specification of cell fate within the neural tube. Sonic hedgehog (SHH) is secreted from the ventral regions of the neural tube, whereas bone morphogenetic protein (BMP) and WNT proteins are secreted from the dorsal regions [18,19,20,21]. These morphogen gradients specify different neuron types along the dorsal-ventral axis, while neural fates commitment along the anterior-posterior axis is modulated by various fibroblast growth factors (FGFs) and retinoic acid (RA) [22,23,24]. These signaling pathways can be modulated by small molecules and/or growth factors to direct iPSCs into neuronal cell fates. For instance, Lorenz Studer’s group first identified that inhibition of SMAD signaling with the noggin protein and the small molecule inhibitor SB431542 increased the efficiency of neural differentiation of both human ESCs and iPSCs into NPCs [25]. Building on this work, iPSC-derived NPCs can be further differentiated into various neuronal subtypes, such as spinal cord motor neurons by treatment with SHH, RA, and FGFs [26,27,28]. On the other hand, forebrain cortical neurons have been produced by treating iPSCs with antagonists of the SHH pathway or combining dual-SMAD inhibition and WNT inhibition with anti-posteriorizing factors, such as Dickkopf-related protein 1 (DKK1) [29,30,31]. In addition, SHH, FGF8, and WNT1 can promote the specification of midbrain neurons, while activation of BMP/SMAD was also reported to promote neurogenesis of midbrain dopaminergic neurons in iPSCs [32,33,34,35].

PSCs also provide a valuable cellular source to generate in vitro retinal cells. The mammalian eye is formed as an evagination from the diencephalon, the region of the neural tube that gives rise to posterior forebrain structures. During development, inhibition of the WNT/BMP signaling pathways are required for forebrain development [36,37], which is crucial for retinal formation as the eye derives from the developing forebrain [38,39]. Furthermore, insulin-like growth factor-1 (IGF-1) specifically promotes eye induction in the *Xenopus* embryos [40]. Based on this knowledge, Thomas Reh’s group described an improved differentiation protocol by treating human ESCs-derived embryoid bodies with DKK-1, noggin, and IGF-1 to generate retinal progenitor cells (RPCs) that could be further differentiated into inner retinal neurons [41]. Building on this work, our group has further developed a multi-stage differentiation protocol, together with magnetic-activated sorting (MACS), to enrich RGCs [42]. Similar enrichment strategy for RGCs was also described by other groups [43,44]. 

Similarly, photoreceptors can be generated from adherent PSC-derived embryoid bodies by inhibition of the WNT/BMP signaling pathways with noggin and DKK-1 [45,46]. The efficiency for photoreceptor differentiation was further improved in a follow-up study using a three-step photoreceptor differentiation protocol [47]. More recently, Zhu et al. reported the generation of photoreceptors from clinical-grade iPSCs, which can functionally integrate into the mouse retina upon transplantation [48]. Likewise, RPE cells can also be generated using human PSCs by removal of bFGF from the culture medium [49,50,51,52]. Subsequent studies have improved the yields and kinetics of RPE differentiation by addition of a range of small molecules and defined factors [53,54,55,56,57]. These approaches give rise to induced RPE within 40–60 days, with classic hexagonal morphology, marker expression, pigmentation, and phagocytic activity.

In addition to adherent differentiation culture, the recent development of organoids provided an exciting 3D culture system for retinal differentiation. These retinal organoids broadly recapitulate in vivo retinogenesis and retinal morphology, exhibiting appropriate apical-basal polarity and development of major cell types into the laminated structure. In a landmark study, Eiraku et al. reported that quick reaggregation of dissociated ESCs under differentiation conditions generates multilayer retinal organoids. Surprisingly, these organoids demonstrated time-dependent self-patterning of all major retinal cell types resembling that of the in vivo retina. Further Notch inhibition at the later stage of differentiation promoted generation of photoreceptors [58,59]. Subsequent improvements of differentiation protocols generated photoreceptors with rudimentary outer-segment discs and light sensitivity, supporting the functional maturation of photoreceptors derived by this organoid method [60,61,62,63,64]. Further maturation of the photoreceptors was achieved in a modified 2D/3D differentiation protocol, generating retinal organoids with photoreceptors bearing outer and inner segments, connecting cilia, and presynaptic structures [65,66]. Notably, the differentiated photoreceptors were able to incorporate into the mouse retina following transplantation, supporting the potential application of PSC-derived retinal organoids for treating retinal degeneration. In summary, the conventional strategy to mimic the neural developmental pathways, using small molecules or recombinant growth factors, have achieved reasonable success in directing neural and retinal differentiation of PSCs.

## 4. Neuronal Differentiation of iPSCs Using Transcription Factors

Transcription factor-based direct differentiation provides an alternative approach to efficiently convert iPSCs into specific neuron types of interest. Compared to differentiation methods using small molecules/growth factors, the use of transcription factors often promote differentiation with faster kinetics, which in turn reduce the cost of cell production. Pang et al. demonstrated that hiPSCs can be directed to become induced neurons (iNs) using a combination of transcription factors, *ASCL1*, *POU3F2*, and *MYT1L* [67]. Strikingly, forced expression of these three transcription factors reprograms the hiPSCs into neurons in a short period of time, exhibiting bipolar neuron-like morphologies as early as day 3 of differentiation. Neuronal markers, such as Class III β-tubulin and MAP2, were detected by day 8, while action potentials were manifested as early as day 6. The rapid conversion of neuronal fate demonstrated a faster path of transcription factor-based neural induction compared to conventional differentiation using growth factors or signaling molecules. Subsequently, other groups have reported different transcription factor cocktails that can promote differentiation towards specific neuronal subtypes. *ASCL1* has emerged as a potent neural reprogramming factor, with its combined expression with other factors determining different neuronal cell fates. Co-expression of *ASCL1*, *NR4A2*, and *LMX1A* promote neuronal reprogramming to dopaminergic neurons [68], whereas co-expression of *ASCL1* and *DLX2* gave rise to GABAergic neurons [69]. On the other hand, sole expression of other widely used pioneer factors, such as *NEUROG2* or *NEUROD1*, has been shown to convert hiPSCs into functional iNs with nearly 100% yield and purity in less than two weeks [70,71]. The use of synthetic mRNA to deliver these transcription factors also provides a rapid, footprint-free method for neuronal reprogramming of iPSC [72]. Further combination of *LHX3* and *ISL1* with *NEUROG2* give rise to more specific hiPSC-derived motor neurons, highlighting the high efficiency and robustness of transcription factor-based direct differentiation [73].

Previous studies have also demonstrated the use of transcription factors to direct iPSC differentiation into retinal neurons. Overexpression of *Pax6* has been shown in mouse iPSCs to push undifferentiated cells first into RPCs, followed by subsequent differentiation into a mixed population of putative RGCs and photoreceptors [74]. A recent report also demonstrated the use of DKK1, noggin, and Lefty A to first differentiate human iPSCs into RPCs, followed by overexpression of *ATOH7* to further induce differentiation into RGCs [75]. This study highlighted the potential of combining small molecules and transcription factors to direct a step-wise retinal neuronal differentiation. In summary, PSCs can be efficiently differentiated into various neuronal subtypes using growth factors, small molecules, or transcription factors. Future studies in improving the robustness, efficiency, and purity of the derived retinal neurons would greatly facilitate their use for tissue engineering and cell therapy.

## 5. Direct Reprogramming to Generate Neurons Using Transcription Factors

Direct reprogramming offers an alternative route of lineage conversion that utilizes transcription factor expression to allow a direct change of cellular identity and bypass most developmental stages. Compared to iPSC, a major advantage of direct reprogramming is that it bypasses the procedure of resetting somatic cells into a pluripotent state, thus reducing the time needed to derive neurons. Previous studies by the Wernig lab showed that the combination of transcription factors *POU3F2*, *ASCL1*, and *MYT1L* transforms both mouse and human fibroblasts into glutamatergic and GABAergic neurons [67,76]. Further work showed that the proneural factor *ASCL1* alone was able to guide the conversion of human fibroblasts into a mix of glutamatergic and GABAergic neurons [77]. Interestingly, *ASCL1* promoted reprogramming of astrocytes to mainly GABAergic neurons [78]. This highlighted that the effect of transcription factor to reprogram cell fate is partly dependent on the starting cell type. A number of transcription factor combinations have been identified for neuronal reprogramming in a range of somatic cell types. For example, astrocytes can also be reprogrammed into neurons using *NEUROG2*, *NEUROD1*, or *SOX2* individually [79,80,81]. Human retina-derived fibroblasts have been induced into neuronal cells expressing mature neuronal markers, functional synapses, and electrophysiology by the combined expression of *ASCL1* and *PAX6* [82]; human pericytes have also been converted into neurons by expression of *ASCL1* and *SOX2* [83], and more specific cholinergic neurons by expression of *ASCL1*, *MYT1L*, *POU3F2*, *TLX3*, and a microRNA miR-124 [84]. Other non-fibroblastic cell sources, such as human T cells or cord blood cells, have also been successfully reprogrammed into iNs using different combinations of factors [85,86]. Collectively, these studies supported the feasibility of direct neuronal reprogramming from a range of different starting cell types.

Generation of specific neuronal subtypes is important to improve modeling and advance cell therapy for neurodegenerative diseases. As the master regulators for neuronal specification, *ASCL1* and *NEUROG2* are commonly used as the core factors and supplemented with other factors to promote neuronal reprogramming to specific subtypes. Additional expression of *LMX1A* and *FOXA2* with *POU3F2*, *ASCL1*, and *MYT1L* directs conversion of human fibroblasts toward dopaminergic neurons [87], while a combination of fewer transcription factors—*ASCL1*, *NR4A2*, and *LMX1A*—also generates functional dopaminergic neurons from both mouse and human fibroblasts [88]. On the other hand, *NEUROG2* can promote generation of cholinergic neurons from human fibroblasts with the help of two small molecules, forskolin, and dorsomorphin [71,72]. Serotonergic neurons can be generated from human fibroblasts by overexpression of *NKX2-2*, *FEV*, *GATA2*, and *LMX1B* in combination with *ASCL1* and *NEUROG2* [89], or by an alternating set of transcription factors—*ASCL1*, *FOXA2*, *FEV*, and *LMX1B* [90]. Interestingly, the conversion efficiency and yield of these serotonergic neurons were significantly increased by p53 knockdown and hypoxic culturing condition [90], indicating a link between p53 signaling, oxidation, and the reprogramming process.

A range of other transcription factor combinations have also been demonstrated to promote lineage conversion into neurons with specialized function, for example, co-expression of *POU4F1* with either *NEUROG1* or *NEUROG2* direct human and mouse fibroblasts into neurons with properties of peripheral sensory neurons, displaying expression of TrkA, TrkB, or TrkC receptors and electrophysiological activity [91]. A combination of five factors, *ASCL1*, *MYT1L*, *NEUROG1*, *ISL2*, and *KLF7*, gives rise to nociceptive neurons expressing specific receptors and channels, such as TRPA1, TRPM8, P2X3, and SCN10A [92]. In addition, excitatory cortical neurons exhibiting electrophysiological properties and key cortical neuronal markers were generated from human fibroblasts by overexpression of *POU3F2*, *MYT1L*, and *FEZF2*. Notably, these iNs displayed a similar molecular signature to fetal cortical neurons and were able to integrate and formed synaptic connections upon transplantation ex vivo into organotypic cultures of adult human cerebral cortex [93].

In the retina field, direct reprogramming technology has been used in the generation of photoreceptors, RPE, and RGCs. The transcription factors *CRX*, *RAX*, and *NEUROD1* were used to convert human iris cells into photoreceptor-like cells with expression of rhodopsin, S opsin, and M/L opsins [94]. Subsequent studies from the same group showed that the same combination of factors can direct reprogramming into photoreceptor-like cells from human dermal fibroblasts [95] or peripheral blood mononuclear cells [96], providing more accessible cell sources compared to iris cells. Interestingly, addition of *OTX2* further improved this reprogramming process, resulting in higher expression of photoreceptor-specific markers, such as S opsin, recoverin, S-arrestin, CNGB3, and PDE6C [95]. Similarly, *NEUROG1* can also reprogram human RPE cell lines into photoreceptor-like neurons, with a range of marker expression including RBP3, recoverin, arrestin, transducin α-subunit, CRX, and L/M opsin [97]. 

On the other hand, Müller glia represents a promising target for direct reprogramming into retinal neurons, as they are the major glial cells in the retina that harbor evolutionary conserved stem and progenitor potentials. Overexpression of *ASCL1* in postnatal Müller glia promoted reprogramming into a pool of iNs with properties of amacrine, bipolar cells, and photoreceptors [98]. Moreover, overexpression of *NEUROG2* can reprogram postnatal Müller glia into iNs with increased level of RGC marker genes, such as *POU4F1*, *SLC17A6*, *CALB2*, *SYN1*, and *PVALB* [98]. Altogether, these studies showed the potential of using transcription factors to drive direct reprogramming into various retinal cell types. 

## 6. Alternative Direct Reprogramming Approaches Using microRNA and Small Molecules 

Besides transcription factors, the use of small molecules and/or microRNA represents an alternative neuronal reprogramming approach. Application of small molecules can facilitate lineage conversion by modulating developmental signaling pathways. For instance, a chemical cocktail of seven small molecules (valproic acid, CHIR99021, RepSox, forskolin, SP600125, GO6983, and Y-27632) was shown to successfully convert human fibroblasts into neuronal cells [99]. Similarly, Wan et al. also reported the use of five chemical cocktails (valproic acid, CHIR99021, DMH1, RepSox, forskolin, Y-27632, and SP600125) to reprogram fibroblasts into neurons [100]. Other non-fibroblastic cell sources were also tested for chemical reprogramming. Human fetal astrocytes were reprogrammed into glutamatergic and GABAergic neurons by four small molecules, DAPT, CHIR99021, SB431542, and LDN193189 [101]. Apart from fetal astrocytes, treatment of valproic acid, CHIR99021, RepSox, forskolin, i-Bet151, and ISX-9 also succeed in transforming human adult astrocytes into neurons. More recently, Mahato et al. showed that fibroblasts can be chemically reprogrammed into rod photoreceptors using a set of small molecules—valproic acid, CHIR99021, RepSox, forskolin, SHH, taurine, and RA. Notably, transplantation of the reprogramed photoreceptors resulted in improvement in visual functions in a mouse degenerative model [102], which highlighted the therapeutic potential of this direct reprogramming strategy in treating blinding retinal diseases. 

microRNAs are also reported to promote direct neuronal reprogramming. For instances, miR-9/9∗ and miR-124 are key neurogenic factors which are capable of reprogramming human fibroblasts into neurons when combined with *NEUROD1* [103,104]. Furthermore, miR-9/9∗ and miR-124 can be supplemented with different transcription factors to generate specific neuronal subtypes, such as striatal neurons when combined with *BCL11B*, *DLX1*, *DLX2*, and *MYT1L* [105,106], and spinal cord motor neurons in combination with *ISL1* and *LHX3* [107]. microRNAs also play an important role in directing glial-to-neuron reprogramming. For example, miR-128 can be used in combination with *NEUROD1*, *ASCL1*, and *LMX1A* to reprogram astrocytes into dopaminergic neurons [108], whereas miR-218 together with *Ascl1* and *Lmx1a* can convert neuron-glia antigen 2 (NG2)-expressing glial cells (NG2 glia) into parvalbumin-containing interneurons in mouse in vivo [109]. Collectively, these results support the use of microRNAs to facilitate transcription factor-based neuronal reprogramming into specific neuronal subtypes.

## 7. Opportunities for Stem Cell-based Therapies for Regenerative Medicine

iPSC and direct reprogramming provide two feasible strategies to derive human cells in vitro for the development of cell therapy. A number of clinical trials for iPSC-derived products have been initiated for the treatment of neurodegenerative diseases. In Japan, a Phase I/II clinical trial led by Jun Takahashi is currently testing the efficacy of using iPSC-derived dopaminergic progenitor cells to treat Parkinson’s disease [110,111]. In the eye, there are promising results for early trials of RPE replacement therapy, with several ongoing phase I/II clinical trials for PSC-derived RPE replacement [112,113,114,115]. More recent efforts in this field featured the use of human fetal-derived RPCs to treat retinitis pigmentosa, including a Phase II trial with intravitreal delivery by JCyte [116] and a PhaseI/II trial with subretinal delivery by ReNeuron [117,118]. The success of these trials would open up possibilities for cell therapy as a treatment for neurodegenerative diseases. Similarly, the advent of ESC/iPSCs provides a promising alternative source for photoreceptor transplantation. Recent studies provided evidence for structural and functional integration of PSC-derived photoreceptor precursors into the retinal circuits [48,65,119,120,121,122,123,124,125], supporting the clinically applicability of using PSC-derived photoreceptors for cell therapy. 

In comparison, the development of RGC replacement therapy is less advanced than those for RPE and photoreceptors. Transplantation of primary RGCs can successfully integrate into the retinal circuitry, supporting the feasibility of RGC replacement therapy [126,127]. In addition, preclinical animal studies showed that transplantation of Müller glia-derived RGCs resulted in some degree of functional recovery in RGC injury models [128,129]. However, limited studies have utilized PSC-derived RGCs for transplantation. In an encouraging study, RPCs were extracted from hESC-derived retinal organoids and transplanted into the vitreous cavity of a mouse model with RGC injury, where the transplanted cells migrated and integrated into the ganglion cell layer in the host retina [130]. Further development to upscale generation and purification of the stem cell-derived retinal cells would facilitate new treatment for retinal regeneration. In particular, transplantation of retinal organoids provided an interesting strategy for cell therapy, as highlighted by recent studies to improve vision in a rodent model with retinal degeneration [131]. 

## 8. Potential of Using In Vivo Reprogramming for Neuroregeneration

The emergence of in vivo reprogramming provided an exciting strategy to promote tissue regeneration, by converting residential cells to replace cells that are lost in diseases or injury. Guo et al. showed that overexpression of *NeuroD1* can directly reprogram reactive glial cells in the cortex into functional neurons in mouse models for Alzheimer’s diseases or stab-injury [132]. Interestingly, astrocytes were mainly reprogrammed into glutamatergic neurons, while NG2 glia were reprogrammed into a mix of glutamatergic and GABAergic neurons. Importantly, the reprogrammed neurons exhibited synaptic responses, supporting the use of in vivo reprogramming to repair neurodegeneration in the cortex [132]. A number of transcription factors have also been tested for neuronal reprogramming in vivo. For instance, *Ascl1*, *Pou3f2*, and *Myt1l* can convert astrocytes into neuron-like cells in the mouse brain [133], while *Ascl1* alone is capable of reprogramming astrocytes into functional neurons in young animals [134]. *Neurog2* has been reported to reprogram astrocytes into neurons in the adult rat brains when combined with FGF2 and EGF treatment, albeit at low efficiency [135]. The addition of *Bcl2* with *Neurog2* also significantly increased the astrocyte-to-neuron reprogramming efficiency [136]. Besides astrocytes, NG2 glia can also be reprogrammed into neuronal cells upon ectopic expression of *Sox2* in a stab injury model [137]. These studies support the feasibility of neuronal regeneration using an in vivo reprogramming approach. 

The application of in vivo reprogramming to promote retinal regeneration is currently a topic that attracts enormous interests. Early studies showed that inactivation of *Nrl* can promote direct reprogramming of rod photoreceptors into cone-like cells, resulting in functional rescue of retinal degeneration in a mouse model of retinitis pigmentosa [138,139]. A subsequent study further showed that this strategy can improve visual responses in three different retinal degenerative models [140]. These studies pave the way for the development of in vivo reprogramming approaches to treat retinal degenerative diseases. 

Recently, a number of high-profile studies identified Müller glia as a potent target for in vivo reprogramming into various retinal neurons. Tom Reh’s group showed that *Ascl1* can successfully reprogram endogenous Müller glia in mice into amacrine, bipolar cells, and photoreceptors in an injury setting [141]. However, the regenerative response to injury was more pronounced in Müller glia in young mice, suggesting a more restricted regenerative capacity of adult mice. Loss of neurogenic capacity in mature Müller glia might be a result of reduced chromatin accessibility. Building on this work, the same group showed that supplementation of a histone deacetylase inhibitor together with *Ascl1* can successfully reprogrammed endogenous Müller glia into functional retinal neurons in adult mice [142]. These studies provided strong evidence to support the feasibility of using Müller glia reprogramming to stimulate retinal regeneration. A subsequent study showed that endogenous Müller glia can be reprogrammed into rod photoreceptors in healthy retina [143]. The authors utilized a two-step strategy, which included β-catenin-induced Müller glia proliferation, followed by reprogramming into rod photoreceptors using *Otx2*, *Crx*, and *Nrl*. Notably, the reprogrammed rod photoreceptors in vivo can functionally integrate into the retinal and visual cortex circuits [143], providing a critical step towards restoring vision by photoreceptor regeneration. More recently, Zhou et al. exploited the RNA-targeting CRISPR system, CasRx, to knockdown *Ptbp1* gene in Müller glia in mouse, resulting in neuronal reprogramming to generate functional RGCs in vivo [144]. The authors showed that the reprogrammed RGCs establish projections toward the dorsal lateral geniculate nucleus and the superior colliculus, and can improve vision functions in a mouse model with retinal injury [144]. Interestingly, the group also showed that *Ptbp1* knockdown in the striatum also promoted astrocyte reprogramming into dopaminergic neurons and improved functional recovery in a mouse model of Parkinson’s disease. These studies collectively support the feasibility of glia-to-neuron reprogramming to regenerate neurons in vivo, paving the path for future therapeutic applications for degenerative diseases affecting the central nervous system (CNS) and the retina.

## 9. Gene Delivery System for Neuronal Reprogramming

The success of gene therapy depends greatly on appropriate gene delivery systems for introducing therapeutic genetic materials into the target cells. Recent advances in viral vector engineering, delivery, and safety have placed viral-based therapy at the forefront of the field of regenerative medicine. Adenoviruses possess high transduction efficiency in a broad range of host cells and have been commonly used as vectors in a large number of studies. Furthermore, adenoviruses do not integrate into the host genome, leading to relatively transient but safe transgene expression. However, high immune responses against adenoviruses limit their therapeutic efficacy for CNS gene therapy [145]. On the other hand, adeno-associated viruses (AAVs) have been applied extensively in clinical trials for neurodegenerative diseases. Indeed, the first FDA-approved retinal gene therapy uses an AAV2 vector [146]. AAV2 has a well-established safety profile in humans and long-term expression in neurons [147,148,149]. Additionally, the ability of AAV9 and AAVrh.10 to penetrate the blood–brain barrier facilitates gene delivery and transduction in the CNS [150,151,152]. Interestingly, a recently engineered AAV-PHP.B was reported to transduce the majority of astrocytes and neurons across multiple CNS regions with an efficiency of at least 40-fold greater than that of the AAV9 capsids [153], demonstrating the potential of engineered AAV vectors for therapeutic applications.

In contrast to AAVs and adenoviruses, lentiviruses are able to fully integrate DNA into the host genome through reverse transcription, thus providing a more stable and long-lasting transgene expression in vivo. As lentiviral vectors are able to package larger transgenes, they were utilised in a clinical trial has utilized the usage of a lentiviral vector for patients with advanced Parkinson’s disease [154]. Overall, viral vectors provide substantial flexibility and a range of therapeutic options that make them potentially useful in a wide range of human diseases.

Apart from viral-based gene delivery systems, synthetic mRNAs have also been investigated for therapeutic proteins expression and gene editing. Previous reports showed that transfection using lipid or polymeric nanoparticles successfully deliver synthetic mRNAs into various cell types [155,156,157], allowing transient production of encoded proteins with a lower risk of insertional mutagenesis. Although clinical applications of synthetic mRNAs in the nervous system are limited thus far, continued advances in mRNA manufacturing and intracellular delivery methods may ultimately lead to the use of mRNA for the treatment of a wide range of neurodegenerative diseases.

## 10. Challenges and Future Direction for Neuronal Reprogramming 

To facilitate clinical application of neuronal reprogramming and stem cells for cell therapy, we need to overcome several major hurdles. Firstly, derivation of patient-specific iPSC for autologous cell therapy is extremely costly, which would impact the availability of the treatment to many patients. To address this, stem cell banks can be set up to provide human leukocyte antigen (HLA)-matched iPSCs. In addition, advances have been made in the development of universal donor iPSC by knocking down genes required for immune recognition, such as HLA class I and II proteins [158]. Secondly, cell therapy often requires a high number of transplanted cells to achieve therapeutic effects, therefore upscaling the manufacturing of iPSCs, as well as their differentiated derivatives, would be important. The use of automated culture systems can improve cell production [159]. In addition, the development of differentiation methods with improved efficiency, as well as the incorporation of cell purification or enrichment strategies (i.e., flow cytometry), would help address this issue. Thirdly, the risk of tumor formation using iPSC-derived cells for transplantation needs to be carefully managed. Rigorous quality control of iPSC-derived cell products would be essential for the development of safe and effective stem cell-based cell therapy.

On the other hand, in vivo reprogramming of endogenous glial cells represents an exciting approach for neuronal regeneration. However, given that extensive levels of neuronal reprogramming are likely required to provide a therapeutic effect in diseases or injuries, there is a risk that in vivo reprogramming may lead to depletion of the endogenous glial cells targeted for reprogramming. Incorporation of strategy to stimulate glial cell proliferation during in vivo reprogramming could be a key to this issue [143]. Further refinement of in vivo reprogramming methods, using optimal reprogramming factor combination and gene delivery system, to improve on efficiency and specificity would be crucial. The development of computational algorithms to predict transcription factors for direct reprogramming provide an exciting tool to facilitate this research, as discussed previously [160]. Secondly, for inherited neurodegenerative diseases it is important to note that the regenerated cells stimulated by in vivo reprogramming would still harbor the disease-causing mutations. Future studies that utilize combinatorial gene therapy to correct genetic mutations together with in vivo reprogramming would be an interesting research direction in the field. Finally, it is important that the regenerated cells stimulated by in vivo reprogramming are carefully characterized for cell identity and functions to ensure the safety of this regenerative strategy.

## 11. Summary and Conclusion

Technological advances in cell reprogramming technology over the past few years have opened up new opportunities for regenerative medicine targeting the central nervous system and the retina. The feasibility of in vitro derivation, using iPSC or direct reprogramming, as well as in vivo reprogramming to generate specific neurons, provide exciting regenerative strategies to repair the nervous system in disease and injury settings. Previous studies have highlighted a range of transcription factors, small molecules, and microRNA in controlling cell fates, which led to the development of new neuronal reprogramming and differentiation methods with better efficiency and specificity. We expect further understanding of the signals involved in development and cell fate determination would advance the field of cellular reprogramming. Future studies to examine the therapeutic potential of transplantation of in vitro cells, as well as in vivo reprogramming, will be an exciting research direction to advance the development of novel neuroregenerative strategies.
